# Mutual positive effects between shrubs in an arid ecosystem

**DOI:** 10.1038/srep14710

**Published:** 2015-09-30

**Authors:** Reyes Tirado, Kari Anne Bråthen, Francisco I. Pugnaire

**Affiliations:** 1 Estación Experimental de Zonas Áridas, Consejo Superior de Investigaciones Científicas, Ctra. Sacramento s/n, La Cañada, E-04120 Almería, Spain; 2 Department of Arctic and Marine Biology, UiT, The Arctic University of Norway, NO-9037 Tromsø, Norway

## Abstract

One-way facilitation in plants has been found in many harsh environments and their role as structural forces governing species composition in plant communities is now well established. However, reciprocal positive effects benefiting two interacting species have seldom been reported and, in recent reviews, conceptually considered merely as facilitation when in fact there is room for adaptive strategies and evolutionary responses. We tested the existence of such reciprocal positive effects in an arid environment in SE Spain using spatial pattern analysis, a species removal experiment, and a natural experiment. We found that the spatial association between *Maytenus senegalensis* and *Whitania frutescens*, two shrub species of roughly similar size intimately interacting in our community, resulted in mutual benefit for both species. Benefits included improved water relations and nutritional status and protection against browsing, and did occur despite simultaneous competition for resources. Our data suggest two-way facilitation or, rather, a facultative mutualism among higher plant species, a process often overlooked which could be a main driver of plant community dynamics allowing for evolutionary processes.

Positive interactions among plants have frequently been reported in the last 20 years^1^,^2^ and many examples are now documented in very different communities and climates, from deserts to arctic environments often involving partners of different size, growth form, or functional type^3^,^4^,^5^. Plants are able to alter environmental conditions and their interactions usually encompass a variety of effects^6^,^7^. For instance, plants buffer climate extremes by shading^4^, can increase soil water available to neighbors through hydraulic lift^8^,^9^, increase soil nutrients beneath the canopy^3^,^10^, or protect from herbivory^11^,^12^,^13^. Such positive effects occur despite competition for resources^14^ and are context-dependent^15^,^16^,^17^. The same pair of species can be mutualists, commensals, or even parasites under different conditions^18^, the “positive” term in the interaction being just one end of a continuum of possible outcomes^6^ that could stretch to mutualism. Although interspecific mutualism represents one of the most important and widely addressed interactions in ecology^18^,^19^,^20^,^21^, plant-plant mutualistic interactions have been guessed^18^ but seldom reported. There is, however, some evidence pointing to such interactions. Pugnaire *et al.*^10^ evidenced a mutualistic interaction between *Retama sphaerocarpa* shrubs and *Marrubium vulgare*, a smaller shrub growing in its understory. When growing together, both shrubs had higher productivity, more leaf N, and overall better physiological status than growing isolated. Holzapfel and Mahal^22^ also found in the Mojave Desert that the community of annual species growing under *Ambrosia dumosa* shrubs had positive effects on the shrub water status, growth, and reproductive output while annual species simultaneously benefited from shrub presence improving survival, biomass production, and seed production. However, despite such empirical evidence, mutually positive interactions between higher plant species have not attracted much attention. Several reasons explain why they may be underreported. One is that few mutualisms involve species occupying the same trophic level^23^, a point particularly hard to assume in sessile organims such as plants sharing similar resources. Mutualism is generally found between different trophic levels (plant-insects, animal-bacteria, algae-fungi) and even at the intraspecific level (e.g., social organisms) but there are very few examples of mutualism within the same trophic level. Another reason is that the concept of mutualism is often restricted to interactions showing specificity and long evolutionary history. Finally, the unsconspicuous nature of such interactions among plants could contribute to this lack of attention. Let us remind that facilitation, now widely acknowledged as a main driver of community dynamics, took decades to become recognized^7^,^24^.

Aware of the possibility that two plants growing together may have effects benefitting both of them –leading to bi-directional facilitation or muatulism–, we addressed the interaction between two shrub species of similar size that intimately interact in semiarid environments in southeast Spain. *Maytenus senegalensis* (Lam.) Exell. subspp. *europaeus* (Celastraceae) is a tall, thorny, evergreen shrub found in coastal zones in SE Spain where it forms characteristic communties within its canopy, locally known as ‘artineras’. *Whitania frutescens* Pau (Solanaceae) is a tall, drought-deciduous shrub of African origin found also in more mesic sites in SE Spain. Individuals of *W. frutescens* (*Whitania* hereafter) in these patchy communities can grow isolated or within the canopy of the dominant species, *M. senegalensis* (*Maytenus* hereafter). We tested whether the observed spatial association between the two species was statistically significant and explored the nature of the interaction between both species through observational and manipulative experiments. We hypothesized that 1) each species will be able to modify the growing conditions for the other; 2) there is competition for resources but, overall 3) both species benefit from growing together.

## Results

**Figure 1:**
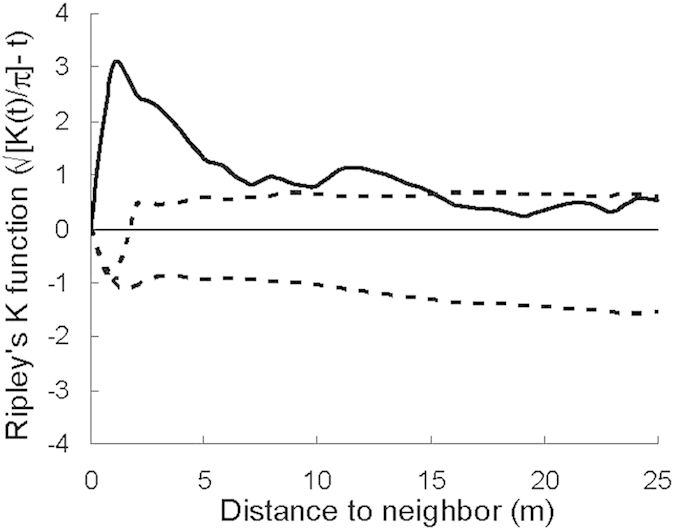
Second-order spatial analysis of the distribution pattern of *Maytenus senegalensis* and *Whitania frutescens* in an ‘artinera’ shrubland in El Ejido (Almería, Spain). The plot of the derived statistics of Ripley’s *K* function (√*K*[(*t*)/π]−t) versus neighbor distance (*t*) reveals spatial patterns at various values of the neighborhood distance *t*. Positive values indicate aggregation, while negative ones signify regularity. Dotted lines give 95% confidence intervals for complete spatial randomness (resulted from 1000 randomizations of actual data). Data represent means calculated from three plots.

**Figure 2:**
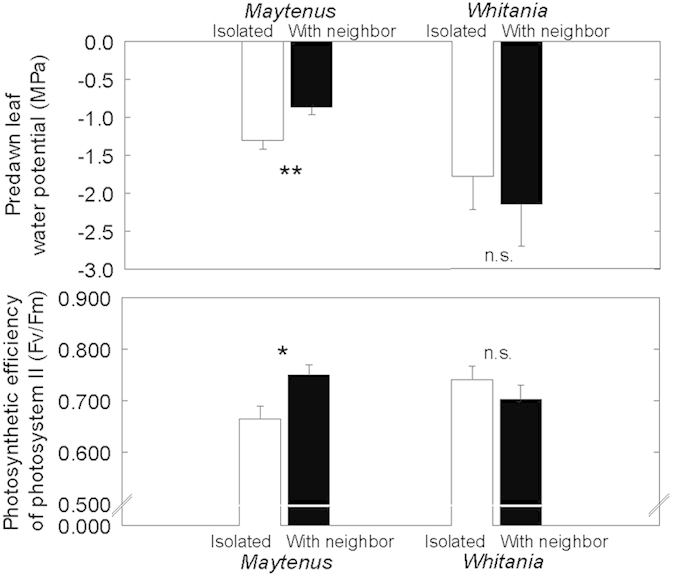
Predawn leaf water potentials and predawn photosynthetic efficiency of photosystem II (F_v_/F_m_) of *Maytenus* and *Whitania* shrubs in spring. Clear bars represent isolated individuals, and solid bars plants with neighbors. N = 4–6 shrubs. Data are mean ± 1 SE; statistically significant differences (U Mann-Whitney test) noted by **(*P* < 0.01) and *(*P* < 0.05).

Second order spatial analysis evidenced a strong association between *Maytenus* and *Whitania* shrubs for distances between 1 and 15 meters (Fig. 1) showing the two species are associated with one another. Plant physiological status depended on neighbour removal, species, and season. Overal predawn water potentials (*Ψ*_pd_) considerably decreased from winter to spring in both species but the decrease was more pronounced in *Whitania* (Table 1). In winter, *Maytenus* and *Whitania* –either isolated or with neighbors– did not differ in *Ψ* or photosynthetic efficiency of photosystem II (F_v_/F_m_). In spring, water status of *Whitania* was unaffected by the presence of *Maytenus*, but *Ψ* of *Maytenus* was higher in plants living with *Whitania* than in isolation, both predawn (Fig. 2) and midday (*Ψ*_isolated_ = −2.14 ± 0.22 *vs*. *Ψ*_+*Whitania*_ = −1.09 ± 0.30, *P* = 0.033). In spring, predawn F_v_/F_m_ was also higher in *Maytenus* living with *Whitania* (Fig. 2) but similar at midday (*Maytenus*: F_v_/F_m isolated_ = 0.646 ± 0.022 *vs.* F_v_/F_m+*Whitania*_ = 0.673 ± 0.035, *P* = 0.62); *Whitania* shrubs living with and without *Maytenus* did not differ in F_v_/F_m_ (Fig. 2). Overall, our data show that there was no detectable influence between neighbors in winter regarding water status and fluorescence emmission (an indication of stress), while in spring *Maytenus’* physiological status benefited from growing with *Whitania,* as shown by its improved water potential and fluorescence.

**Figure 3:**
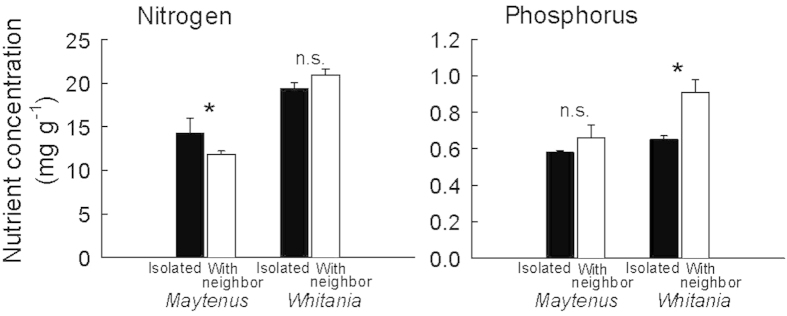
Nitrogen and phosphorus leaf concentration in *Whitania* and *Maytenus* shrubs growing isolated (clear bars) or with neighbors (solid bars). n = 3. Data are mean ± 1 SE; statistically significant differences at *P* < 0.05 (U Mann-Whitney test) noted by *.

*Whitania* shrubs living isolated did not differ from plants living with *Maytenus* regarding leaf N content, but leaf P was higher in plants living with *Maytenus* (Fig. 3). Conversely, N content in *Maytenus* leaves was highest in plants living with *Maytenus*, while P did not differ between treatments (Fig. 3).

**Figure 4:**
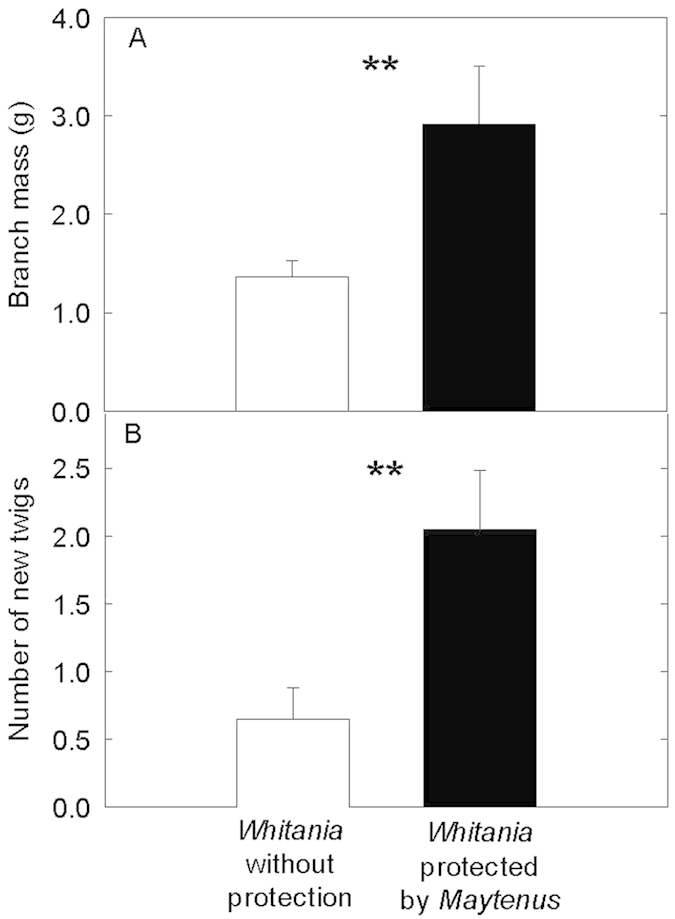
Dry mass of branches (A) and number of new twigs per branch (B) of *Whitania* plants without protection and protected by thorny shrubs. N = 20 *Whitania* plants. Data are mean ± 1 SE; statistically significant differences (U Mann-Whitney test) noted by **(P < 0.001).

**Figure 5:**
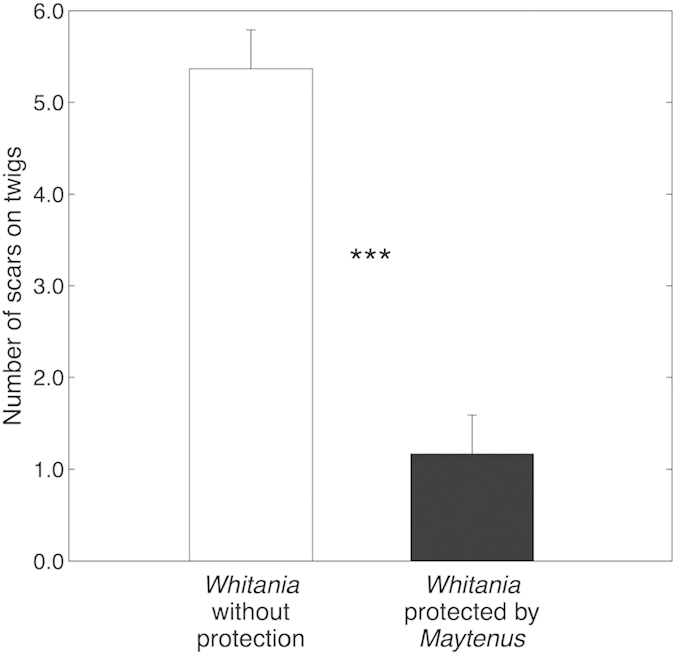
Number of scars on twigs of *Whitania* individual plants (mean ± 1 SE) without protection and protected by thorny *Maytenus* shrubs. N = 20 plants. Data are model estimates from linear mixed effect model; statistically significant differences are noted by ***(p < 0.0001).

*Whitania* shrubs living isolated in gaps were strongly affected by browsing. Most sampled branches in isolated shrubs had been eaten and lost nearly 50% of their mass (Fig. 4A), being shorter (16.6 ± 1.1 cm *vs* 24.7 ± 2.0 cm, *P* < 0.01) and with less twigs than protected branches (Fig. 4B). Differences in branch width at the base were not statistically significant (5.34 ± 0.38 mm protected *vs.* 6.20 ± 0.35 mm unprotected; *P* = 0.19) but differences in leaf mass were (0.68 ± 0.10 g *vs.* 0.18 ± 0.03 g; *P* < 0.0001) while woody stem mass did not differ between protected and unprotected branches (2.24 ± 0.51 g *vs.* 1.57 ± 0.42 g; *P* = 0.27). *Whitania* shrubs living isolated in gaps were strongly affected by browsing in comparison to shrubs protected from *Maytenus*, with number of scars on twigs being five times higher (Fig. 5).

## Discussion

The association of different species in patches is a notable feature of arid and semiarid environments often considered indicative of positive interactions^25^,^26^,^27^ but seldom tested^28^,^29^. Our data show that, indeed, there was a signifcant spatial aggregation between *Maytenus* and *Whitania* and an improved performance of individuals living in patches compared to individuals living isolated, suggesting a mutually beneficial interaction. This two-way facilitation was consequence of the interaction between plants rather than effect of environmental factors, as the presence of microsites was not evident in this system. We do not have fitness data and are therefore unable to test the full consequences of this interaction. But we did find that *Maytenus* benefited from the presence of *Whitania* by showing values of water potential and photochemical efficiency of photosystem II higher than isolated plants. Simultanously, *Whitania* benefited from grazing protection provided by the intricate and thorny *Maytenus* canopy.

Water potential of both species declined substantialy from winter to spring, evidencing a shortage in water availability. In winter, water was abundant enough to keep *Ψ* close to zero but in spring, the time of highest growth rate, *Maytenus Ψ*_pd_ and F_v_/F_m_ improved with the presence of *Whitania* (Fig. 2) despite the higher transpiring leaf surface in patches and the likelihood of increased water limitation derived from competition for water. *Whitania* water status, however, was not affected by the presence of *Maytenus* and, if anything, tended to be worse in patches. Water status and photochemical efficiency have a strong impact on overall plant performance^30^ so that the improved physiological status of plants living in patches can likely lead to higher fitness, as is often the case in arid zones^27^.

Patterns of *Ψ* in *Whitania* are consistent with a superficial root system, while *Maytenus Ψ* data suggest it has access to deeper water resources^31^,^32^,^33^. Our data suggest that *Whitania* helps improve *Maytenus* water relations most likely through soil shading or hydraulic lift. Shade reduces thermal amplitudes and decreases soil water evaporation under the canopy^34^, which promotes an increase in soil moisture compared to bare ground and overall, a better water balance for plants^2^,^4^,^35^. Hydraulic lift is common whenever species have a dual root system accessing soil layers differing in water potential^9^,^36^,^37^. Increases in soil moisture linked to reduced evapotranspiration have been identified as a key mechanism driving positive interactions in dry ecosystems^4^,^35^,^38^. In addition, by growing within the *Maytenus* canopy, *Whitania* may increase the boundary layer conductance of the whole canopy, lessening transpirational demand^39^ hence improving overall plant water status.

The contrasting effects on the neighbors’ leaf nutrient content show that close interacting plants may compete for some resources while simultaneously improving availability for others. In our field site *Maytenus* growing with *Whitania* had lower leaf N than isolated individuals, suggesting competition for N (but simultanously displayed higher *Ψ*_pd_ and F_v_/F_m_). Conversely, *Whitania* growing with *Maytenus* had higher leaf P than isolated individuals, pointing to higher P availability under *Maytenus* canopies most likely linked to higher micorrhizal inocula in the understorey. This is often the case in arid environments where micorrhizas play a crucial role^40^ and where vegetation patches act as micorrhiza reservoirs^41^.

Thorny *Maytenus* shrubs provided an effective protection to *Whitania*, a palatable species higly browsed on when growing isolated. Protection from grazing appears as a major benefit for *Whitania* shrubs growing with *Maytenus*, as in other systems under high herbivory pressure^1^,^12^. Examples of associational defences have been widely reported^42^,^43^. In SE Spain, unpalatable *Artemisia barrelieri* shrubs facilitate seed germination and seedling establishment of more palatable *Anthyllis cytisoides* shrubs, providing shelter from herbivory during early growth stages^44^. *Stipa tenacissima* tussocks have also been found to reduce browsing by rabbits^38^,^45^. Alados *et al.*^46^ observed a positive association among palatable and unpalatable species which was more evident for the most palatable species, *Ballota hirsuta*, whose association with unpalatable species increased with grazing pressure. In most cases, benefactor species appear to physically shelter or hide beneficiary species from herbivores^42^,^47^.

Our results suggest that plants interact in a multidimensional space, with synergic or antagonistic effects depending on the factor considered^22^,^35^. Such effects depend on plant traits which have rarely been assessed in the framework of the interaction between beneficiary and benefactor species (but see refs. 48, 49, 50). There are well-known traits that characterize facilitator species^49^, like the ability to fix N, casting a shade^1^,^51^ or buffer temperature extremes^14^,^51^. Thus complementary suites of traits in different species can lead to mutually beneficial interactions, and selection should strongly favour traits promoting benefits from neighbours^20^ particularly in harsh environments^49^,^50^.

Our two species improve conditions regarding specific resources while competed for others with an overall positive balance for both and should have consequences for fitnsess –which we did not measure. Despite previous evidence on reciprocal beneficial effects between different plant species, such results still challenge our current understanding of plant interactions. We suggest that the interaction between *Maytenus* and *Whitania* may be a facultative mutualism, a term which is currently considered to subsume even transient interactions of small effect as long as both partners experience a net positive effect^20^,^23^,^52^. This mutual interaction in our field site is based on herbivory. In a grazed landscape facilitation is likely to promote fitness of palatable species that otherwise would tend to disappear, being able to recruit and maintain a viable population. In other words, facilitation occurred when the species were deviated from their optimum niche^15^,^16^,^17^.

Therefore, complex interactions –which may have been overlooked to date– result on reciprocal positive interactions which influence community structure. This adds to the better-known, one-way positive plant-plant interactions which have proved to be main drivers of plant community dynamics^24^ and evolution^53^.

## Methods

### Field site and species

In winter 1998 a research site was established in a *Maytenus* shrubland in El Ejido (36° 47′ N, 2° 46′ W, 80 m elevation), Almería the best preserved population of this species in Spain. The climate is Mediterranean semi-arid, with a pronounced dry season from June to September with almost no rain in most years. Mean annual precipitation is 280 mm and mean annual temperature 18.5 °C, with January (mean temperature 12.5 °C) and August (mean temperature 28.0 °C) as the coldest and warmest months, respectively. Soils are calcic regosols and cambisols, with a mixed clayey substrate with a calcic hardpan close to the soil surface.

The dominant shrubs *Maytenus* and *Whitania* appear frequently aggregated in clumps where branches are intimately associated but many other species are found in these patches, from shrubs (up to 13 different species in a patch^54^) to forbs and grasses while the space in between is only scantily covered by small shrubs and geophytes. *Maytenus* is a thorny shrub up to 3 m tall, with coriaceous perennial leaves and an intricate spherical canopy. *Whitania* grows up to 2.5 m and has thin leaves that are shed before the dry season. Browsing by sheep and goat is frequent in the area and exposed *Whitania* individuals are preferred fodder, showing smaller and altered canopy shapes.

Both species have fleshy fruits and the spatial association of the two species may be just a consequence of dispersal patterns (the main dispersers are frugivorous birds). However, isolated individuals are frequently observed in more mesic habitats (such as shady canyons or higher elevation sites) suggesting that dispersal is rather random and the co-occurrence of both species has further implications.

### Spatial analysis

Three 50 m × 50 m plots were established in the study site in October 1999. The coordinates of the estimated center of every *Maytenus* and *Whitania* individual taller than 10 cm were recorded to the nearest 5 cm. Spatial distribution patterns were analyzed by using Ripley’s *K* function^55^. The null hypothesis of complete spatial randomness between the two species was tested with a modified procedure for analysis of bivariate distribution patterns, and statistical analysis of the data was performed using the SPPA software^56^. Ripley’s K function is a recommended technique for bivariate point pattern analysis^57^. It considers each plant as the central point of a circle of radius t, counting the number of points found within the circle. We used the weighting approach to correct for edge effects^56^. If the distribution of the points is Poisson random, the expected value of the cumulative function K(t) equals *πt*^*2*^, i.e. the area of a circle of radius *t*. For an easier interpretation, the derived sample statistic √*K*[*(t)/π*]*−t* is plotted, as this expression has a zero expectation for any value of t when the pattern is Poisson random, being positive when it is aggregated and negative when regular (uniform).

### Specific neighbor effect

To investigate the specific effect of *Maytenus* and *Whitania* on each other, in February 1998 a removal experiment was set up. 18 patches similar in physiognomy and size (about 3 m tall, 2 m in diameter) were selected within a 2-ha plot in which spatial analyses were conducted and individuals of either *Maytenus* or *Whitania* were removed in a subset of six randomly selected patches, while another six patches were kept as control. Stems of non-target species were clipped to the base at the onset of the experiment and resprouts were removed frequently.

Two years after removal we measured physiological variables in late winter (February 2000) and at the time of maximum growth in spring (late March 2000) but not in summer because *Whitania* is a summer-deciduous species. Physiological status was assessed by measuring predawn and midday water potential (*Ψ*_pd_) (n = 4–6 per patch) in branch tips, randomly selected at ~1.5 m heigh in the canopy facing east, with a pressure chamber (Skye Instruments, Powys, UK) and predawn and midday photosynthetic efficiency of PS II (F_v_/F_m_) in 30′ dark-adapted leaves (n = 4–6) with a chlorophyll induction fluorimeter (PEA; Hansatech, Kings Lynn, UK).

### Nutrient analysis

We determined nutrient content of *Maytenus* and *Whitania* leaves of individuals living isolated and in manipulated patches. The first mature, fully developed leaf in several branches per individual, always with similar orientation and position in the canopy, were collected, combined to make a composite sample, dried at 70 °C, and analyzed for N and P (n = 3 per treatment and species) by Kjeldahl and colorimetric techniques, respectively.

### Browsing assessment

Livestock browsing by goats and sheeps is frequent in the area and its effect on plants is evident at our field site. We assessed browsing by comparing *Whitania* with and without protection from *Maytenus*. We selected the tallest 1 year-old branch and recorded branch diameter at the base with a digital caliper in 20 *Whitania* shrubs growing under thorny *Maytenus* branches and paired them with another 20 *Whitania* individuals living without protection. Paired individuals were between 1 and 5 m from each other. All individuals were similar in size (canopy diameter between 0.90 and 1.4 m). We sampled plants in May 2001, at the end of the growing season, in an attempt to capture browsing effects after potential growth compensation. In each plant we harvested the selected branch and recorded branch diameter, total length to the closest millimeter, and the number of new twigs. In the laboratory we separated leaves from stems, and plant material was dried at 70 °C for 72 hours and weighed. In addition, we used scars from browsing events on *Whitania* twigs as indicator of browsing intensity, as they were clearly visible. We recorded scars in 20 pairs of *Whitania* shrubs, each pair consisting of a *Whitania* individual growing under thorny *Maytenus* branches and another *Whitania* individual living in a gap. For each *Whitania* shrub we recorded the number of clearly visible scars in three 0.1 × 0.1 m quadrats at 1 m height distributed at angles of 0°, 120° and 240° towards the shrub basal centre. For each shrub the 0° was always south. If a framed area did not contain *Whitania* twigs, the frame was moved to the closest area of the shrub with *Whitania* twigs. Visible scars deeper than 0.2 m from the shrub canopy were not recorded, as such scars were older and less visible.

### Data analysis

Data (but for estimates of browsing intensity) were analyzed with Data Desk 6 (Data Description, Ithaca, New York, USA). Due to heterocedasticity of some variables, data were analyzed by non-parametric U Mann-Whitney tests. Water potential and chlorophyll fluorescence data were tested at the species level for differences between dates (winter *vs.* spring) and between treatments. Values in text and figures represent mean ± 1 standard error.

To analyse estimates of browsing intensity we used a linear mixed effects model^58^ in the R environment, version 3.0.2. Number of scars was used as response variable and *Whitania* with or without protection from *Maytenus* as fixed factor. Random effects were modelled as angle of estimate nested in shrub pair. The model was found satisfactory from standard diagnostics on variance of residuals and outliers.

## Additional Information

**How to cite this article**: Tirado, R. *et al.* Mutual positive effects between shrubs in an arid ecosystem. *Sci. Rep.* **5**, 14710; doi: 10.1038/srep14710 (2015).

## Figures and Tables

**Table 1:** Pre-dawn (*Ψ*_pd_) and mid-day (*Ψ*_md_) leaf water potential of *Maytenus* and *Whitania* shrubs growing alone in winter and spring (n = 8–12).

	Maytenus			Whitania		
	Winter	Spring		Winter	Spring	
*Ψ*_pd_ (MPa)	−0.63 ± 0.08	−1.11 ± 0.10	**	−0.66 ± 0.05	−1.92 ± 0.33	***
*Ψ*_md_ (MPa)	−1.77 ± 0.12	−1.60 ± 0.26	n.s.	−1.36 ± 0.72	−3.20 ± 0.44	***

Data are mean ± 1 SE; statistically significant differences (U Mann-Whitney test) noted by ***(*P* < 0.001) and **(*P* < 0.01).

## References

[b1] Callaway R. M. Positive Interactions and Interdependence in Plant Communities. (Springer, Dordrecht, 2007).

[b2] Callaway R. M. & Pugnaire F. I. Facilitation in plant communities, in Functional Plant Ecology (eds Pugnaire F. I. & Valladares F.) 435–456 (CRC Press, Boca Raton, 2007).

[b3] Vetaas O. R. Micro-site effects of trees and shrubs in dry savannas. J. Veg. Sci. 3, 337–344 (1992).

[b4] Pugnaire F. I., Armas C. & Valladares F. Soil as a mediator in plant-plant interactions in a semi-arid community. J. Veg. Sci. 15, 85–92 (2004).

[b5] Scholes R. J. & Archer S. R. Tree-grass interactions in savannas. Annu. Rev. Ecol. Syst. 28, 517–544 (1997).

[b6] Stachowicz J. J. Mutualism, facilitation, and structure of ecological communities. Bioscience 51, 235–246 (2001).

[b7] Brooker R. W. *et al.* Facilitation in plant communities: the past, the present, and the future. J. Ecol. 96, 18–34 (2008).

[b8] Dawson T. E. Hydraulic lift and water use by plants, implications for water balance, performance and plant-plant interactions. Oecologia 95, 565–574 (1993). 10.1007/BF00317442 28313298

[b9] Prieto I., Armas C. & Pugnaire F. I. Water release through plant roots: new insights into its consequences at the plant and ecosystem level. New Phytol. 193, 830–841 (2012). 22250761 10.1111/j.1469-8137.2011.04039.x

[b10] Pugnaire F. I., Haase P. & Puigdefábregas J. Facilitation between higher plant species in a semiarid environment. Ecology 77, 1420–1426 (1996).

[b11] Hay M. E. Associational plant defenses and the maintenance of species diversity, turning competitors into accomplices. Am. Nat. 128, 617–641 (1986).

[b12] Callaway R. M., Kikodze D., Chiboshvili M. & Khetsuriani L. Unpalatable plants protect neighbors from grazing and increase plant community diversity. Ecology 86, 1856–1862 (2005).

[b13] Gómez J. M. Long-term effects of ungulates on performance, abundance, and spatial distribution of two montane herbs. Ecol. Monogr. 75, 231–258 (2005).

[b14] Pugnaire F. I. & Luque M. T. Changes in plant interactions along a gradient of environmental stress. Oikos 93, 42–49 (2001).

[b15] Liancourt P., Callaway R. M. & Michalet R. Stress tolerance abilities and competitive responses determine the outcome of biotic interactions. Ecology 86, 1611–1618 (2005).

[b16] Gross N., Liancourt P., Choler P., Suding K. N. & Lavorel S. Strain and vegetation effects on local limiting resources explain the outcomes of biotic interactions. Perspect. Plant. Ecol. Evol. Syst. 12, 9–19 (2010).

[b17] Le Bagousse-Pinguet Y., Liancourt P., Gross N. & Straile D. Indirect facilitation promotes macrophyte survival and growth in freshwater ecosystems threatened by eutrophication. J. Ecol. 100, 530–538 (2012).

[b18] Bronstein J. L. Our current understanding of mutualism. Q. Rev. Biol. 69, 31–51 (1994).

[b19] Boucher D. H. The biology of mutualism. (Oxford University Press, New York, 1985).

[b20] Bronstein J. L. The evolution of facilitation and mutualism. J. Ecol. 97, 1160–1170 (2009).

[b21] Pyke D. A. & Archer S. Plant-plant interactions affecting plant establishment and persistence on revegetated rangeland. J. Range Managem. 44, 550–557 (1991).

[b22] Holzapfel C. & Mahall B. E. Bidirectional facilitation and interference between shrubs and annuals in the Mojave Desert. Ecology 80, 1747–1761 (1999).

[b23] Bronstein J. L. Mutualisms, in Evolutionary Ecology: Perspectives and Synthesis (eds Fox C., Fairbairn D. & Roff D.) 315–330 (Oxford University Press, Oxford, 2001).

[b24] Bruno J. F., Stachowicz J. J. & Bertness M. D. Inclusion of facilitation into ecological theory. Trends Ecol. Evol. 18, 119–125 (2003).

[b25] Went F. W. The dependence of certain annual plants on shrubs in southern California deserts. Bull. Torrey Bot. Club 69, 100–114 (1942).

[b26] Haase P., Pugnaire F. I., Clark S. C. & Incoll L. D. Spatial patterns in a two-tiered semi-arid shrubland in southeastern Spain. J. Veg. Sci. 7, 527–534 (1996).

[b27] Tirado R. & Pugnaire F. I. Shrub spatial aggregation and consequences for reproductive success. Oecologia 136, 296–301 (2003). 12695906 10.1007/s00442-003-1264-x

[b28] Silvertown J. & Wilson J. B. Community structure in a desert perennial community. Ecology 75, 409–417 (1994).

[b29] Tirado R. & Pugnaire F. I. Community structure and positive interactions in constraining environments. Oikos 111, 437–444 (2005).

[b30] Ehleringer J. R., Schwinning S. & Gebauer R. Water use in arid land ecosystems, in Physiological Plant Ecology (eds Press M. C. *et al.*) 347–366 (Blackwell Science Ltd., London, 1999).

[b31] Tyree M. T. Water relations and hydraulic architecture, in Functional Plant Ecology (eds Pugnaire F. I. & Valladares F.) 175–211 (CRC Press, Boca Raton, 2007).

[b32] Saha S., Strazisar T. M., Menges E. S., Ellsworth P. & Sternberg L. Linking the patterns in soil moisture to leaf water potential, stomatal conductance, growth, and mortality of dominant shrubs in the Florida scrub ecosystem. Plant Soil 313, 113–127 (2008).

[b33] Armas C. & Pugnaire F. I. Ontogenetic shifts in interactions of two dominant shrub species in a semi-arid coastal sand dune system. J. Veg. Sci. 20, 535–546 (2009).

[b34] Domingo F., Villagarcía L., Brenner A. J. & Puigdefábregas J. Evapotranspiration model for semi-arid shrub-lands tested against data from SE Spain. Agric. Forest Meteor. 95, 67–84 (1999).

[b35] Maestre F. T., Bautista S. & Cortina J. Positive, negative, and net effects in grass-shrub interactions in mediterranean semiarid grasslands. Ecology 84, 3186–3197 (2003).

[b36] Caldwell M. M., Dawson T. E. & Richards J. H. Hydraulic lift: consequences of water efflux from the roots of plants. Oecologia 113, 151–161 (1998). 10.1007/s004420050363 28308192

[b37] Ryel R. J., Caldwell M. M., Leffler A. J. & Yoder C. K. Rapid soil moisture recharge to depth by roots in a stand of *Artemisia tridentata*. Ecology 84, 757–764 (2003).

[b38] Maestre F. T., Bautista S., Cortina J. & Bellot J. Potential for using facilitation by grasses to establish shrubs on a semiarid degraded steppe. Ecol. Appl. 11, 1641–1655 (2001).

[b39] Lambers H., Chapin F. S. III & Pons T. L. Plant Physiological Ecology. 2nd ed. (Springer-Verlag, New York, 2009).

[b40] Martínez-García L. B., Armas C., Miranda J. D., Padilla J. M. & Pugnaire F. I. Shrubs influence arbuscular mycorrhizal fungi communities in a semiarid environment. Soil Biol. Biochem. 43, 682–689 (2011).

[b41] Martínez L. B. & Pugnaire F. I. Arbuscular mycorrhizal fungi host preference and site effects in two plant species in a semiarid environment. Appl. Soil. Ecol. 48, 313–317 (2011).

[b42] Baraza E., Zamora R., Hódar J. A. & Gómez J. M. Plant-herbivore interaction: beyond a binary vision, in Functional Plant Ecology (eds Pugnaire F. I. & Valladares F.) 481–514 (CRC Press, Boca Raton, 2007).

[b43] Smit C., Den Ouden J. A. N. & Muller-Scharer H. Unpalatable plants facilitate tree sapling survival in wooded pastures. J. Appl. Ecol. 43, 305–312 (2006).

[b44] Haase P., Pugnaire F. I., Clark S. C. & Incoll L. D. Spatial pattern in Anthyllis cytisoides shrubland on abandoned land in southeastern Spain. J. Veg. Sci. 8, 627–634 (1997).

[b45] Soliveres S., de Soto L., Maestre F. T. & Olano J. M. Spatio-temporal heterogeneity in abiotic factors modulate multiple ontogenetic shifts between competition and facilitation. Perspect. Plant Ecol. 12, 227–234 (2010).

[b46] Alados C. L., Giner M. L. & Pueyo Y. An assessment of the differential sensitivity of four summer-deciduous chamaephytes to grazing and plant interactions using translational asymmetry. Ecological Indic. 6, 554–566 (2006).

[b47] Bråthen K. A. & Lortie C. J. A portfolio effect of shrub canopy height on species richness in both stressful and competitive environments. Funct. Ecol. in press (2015).

[b48] Schöb C., Butterfield B. J. & Pugnaire F. I. Fundation species and trait-based community assembly. New Phytol. 196, 824–834 2012. 22978646 10.1111/j.1469-8137.2012.04306.x

[b49] Schöb C., Armas C., Guler M., Prieto I. & Pugnaire F. I. Variability in functional traits mediates plant interactions along stress gradients. J. Ecol., 101, 753–762 2013.

[b50] Schöb C., Armas C. & Pugnaire F. I. Direct and indirect interactions co-determine species composition in nurse plant systems. Oikos 122, 1371–1379 2013.

[b51] Moro M. J., Pugnaire F. I., Haase P. & Puigdefábregas J. Mechanisms of interaction between Retama sphaerocarpa and its understory layer in a semi-arid environment. Ecography 20, 175–184 (1997).

[b52] Bronstein J. L. & Holland J. N. Mutualism, in Encyclopedia of Ecology, Vol. 3. (eds Jorgensen S. E. & Fath B. D.) 2485–2491 (Elsevier, Oxford, 2008).

[b53] Valiente-Banuet A., Vital A., Verdú M. & Callaway R. M. Recent Quaternary plant lineages sustain global diversity by facilitating ancient Tertiary lineages. Proc. Natl. Acad. Sci. USA 103, 16812–16817 (2006). 17068126 10.1073/pnas.0604933103 PMC1636537

[b54] Tirado R. Positive interactions between plants in semi-arid communities: mechanisms and consequences. PhD Dissertation (University of Seville, 2003).

[b55] Ripley B. D. The second-order analysis of stationary processes. J. Appl. Probab. 13, 255–266 (1976).

[b56] Haase P. Spatial pattern analysis in ecology based on Ripley’s *K*-function, Introduction and methods of edge correction. J. Veg. Sci. 6, 572–582 (1995).

[b57] Dale M. R. T. Spatial Pattern Analysis in Plant Ecology (Cambridge University Press, Cambridge, 1999).

[b58] Pinheiro J. C. & Bates D. M. Linear mixed-effects models: basic concepts and examples (Springer, New York, 2000).

